# Review of Disordered Eating Behaviors in Cystic Fibrosis

**DOI:** 10.3390/life15091355

**Published:** 2025-08-27

**Authors:** Kate Elizabeth Powers, Allison Bustos, Jacob McCoy, Elizabeth Reid, Erin Scallorn, Jade Robichaud, Amanda S. Bruce

**Affiliations:** 1 Department of Pediatrics, Albany Medical College, Albany, NY 12208, USA; 2 Department of Internal Medicine and Pediatrics, University of Kansas Health Center, Kansas City, KS 66103, USA; amcmullen2@kumc.edu (A.B.); jrobichaud@kumc.edu (J.R.); abruce2@kumc.edu (A.S.B.); 3 Albany Medical College School of Medicine, Albany, NY 12208, USA; mccoyj1@amc.edu; 4 Pulmonary and Sleep Medicine Division, Children’s Hospital of Philadelphia, Philadelphia, PA 19104, USA; reide1@chop.edu; 5 McLane Children’s Clinics, Baylor Scott and White Health, Temple, TX 76502, USA; erin.scallorn@bswhealth.org

**Keywords:** nutrition, chronic disease, appetite, screening, assessment, eating disorders, food choices

## Abstract

Background: CF transmembrane conductance regulator (CFTR) modulators are available for 90% of people with cystic fibrosis (PWCF), which has contributed to substantial nutritional changes. PWCF identify differences in their relationship with food, as well as alterations in body size and image when taking CFTR modulators. This has led to increasing risks relating to issues with body image, disordered eating behaviors (DEBs), and eating disorders (EDs). DEBs can be an early indication of an ED. CF care has traditionally emphasized body mass index and weight gain, which may have heightened the critical focus of body habitus. Prior to CFTR modulators, the “legacy diet” was often promoted and after years of encouragement to eat high volumes of calorically dense foods, PWCF on modulators have shared that the subsequent body changes have been challenging. Given the body changes that PWCF may have experienced, CF care team nutritional guidelines are evolving. The prevalence and etiology of EDs is largely unknown. Therefore, interventions designed to reduce risk factors for EDs and enhance protective factors against the development of DEBs need to be prioritized. To date, there are no reliable and validated screening tools in the United States to identify DEBs for PWCF. The purpose of this paper is to (1) review eating behaviors and disordered eating in PWCF, and (2) discuss important future directions for the assessment and treatment of DEBs to improve quality of life for PWCF.

## 1. Introduction

**Figure 1:**
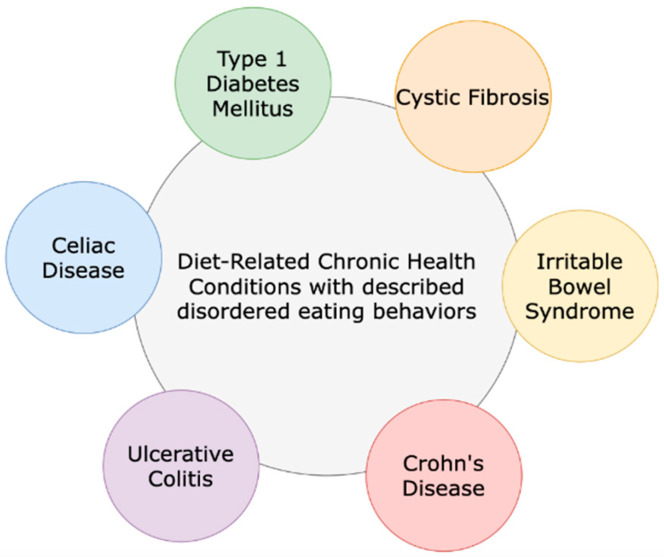
Cystic fibrosis and other diet-related chronic health conditions that include dietary regimen recommendations for treatment and management.

Cystic fibrosis (CF) is a genetic disorder that occurs in all races and ethnicities. CF impacts multiple organ systems of the body and is considered a diet-related chronic health condition (DR-CHC). Chronic health conditions (CHCs) are defined as health conditions with a biological, psychological, or cognitive basis; they are expected to last for at least one year, with symptoms that may limit function and activities, and require medical care or related services. DR-CHCs (Figure 1) include CF, inflammatory bowel disease, type 1 diabetes mellitus, celiac disease, and irritable bowel syndrome and are CHCs with prescribed dietary regimens [1,2]. CF is associated with a spectrum of diseases that can cause progressive bronchiectasis, pancreatic insufficiency, and liver and gastrointestinal dysfunction. There are an estimated 40,000 diagnosed persons living with CF (PWCF) in the United States (U.S.) [3]. With recent advances in treatments for CF, poor nutritional status and low body mass index (BMI) are less common than in the past. Historically, mal- absorption and high-caloric expenditure, in part due to the increased effort of breathing, contributed to poor nutritional status. Now that CF transmembrane conductance regulator (CFTR) modulators are available for approximately 90% of PWCF (based on age and mutation eligibility), the nutritional landscape and outcomes have changed [4].

Many PWCF who take CFTR modulators have experienced significant weight gain, and recent estimates in a U.S. sample show that more than 30% of adults living with CF are in the overweight or obese (OW/OB) range [5]. Estimates in Canada are lower, but adults with CF who can be categorized in the OW/OB range exhibited worse cardio-metabolic profiles compared to adults with CF in the normal weight range [6]. In 2022, the CF Foundation Patient Registry identified that 65% of adults >20 years of age were meeting BMI goals for PWCF, while 13% are now considered in the obese BMI category, as defined by a BMI > 30 kg/m^2^ [7]. Adults with CF in a normal BMI range can exhibit a normal weight obesity, which is significantly higher than the typical body fat percentage and is associated with poorer lung function [4,8]. Adults with CF are also at a greater risk for increased visceral adipose tissue, which has been shown to be associated with elevated fasting glucose levels [9].

While often grateful for personal health improvements, many PWCF identify substantial differences in their relationship with food, as well as significant alterations in their body size and body image when taking CFTR modulators [10]. Many PWCF living with a chronic disease that focuses on food choices, nutritional status, and body size have noticed a considerable change in conversations with care teams in the era of CFTR modulators [10]. In addition, there is very little data on body image in children with CF. This new context has led to the possibility of an increased risk for problems with body image, relationships with food, disordered eating behaviors (DEBs), and, in some cases, an eating disorder (ED) [11]. The purpose of this paper is (1) to review eating behaviors and disordered eating in PWCF and (2) to discuss important future directions for the assessment and treatment of DEBs/EDs to improve the quality of life for PWCF.

As a narrative review, the authors offer a broad overview of disordered eating in PWCF to identify key concepts and current gaps within the field. This narrative review sought to identify English-language articles reporting disordered eating and cystic fibrosis. Published journal articles and articles in press were included. The PubMed/MEDLINE database was initially searched in March 2024, as well as being searched again in January 2025 and July 2025. The search concepts included a variety of keywords to describe disordered eating, cystic fibrosis, and chronic diseases including cystic fibrosis, disordered eating behaviors, maladaptive eating, nutrition, eating disorders, chronic disease, appetite, screening, assessment, and food choices. The authors carefully reviewed the titles of articles and abstract data to extract the most pertinent studies to include in this narrative review. To be included, articles needed to be available in English and be peer-reviewed published academic articles.

### 1.1. Physiology of Appetite

PWCF historically had a different physiology to persons living without a chronic disease. PWCF commonly experience altered absorption and metabolism, along with an increased effort to breathe, often resulting in a caloric deficit, weight loss, and poor nutritional status [11]. The drive to eat, also referred to as appetite, is foundational for life; the complementary state is referred to as satiety and denotes feeling satiated or “full” [12]. The energy balance equation refers to caloric intake and caloric expenditure; it states that the rate of change in energy storage in the body, such as fat mass and fat-free mass, is equal to the rate of energy intake minus the rate of energy expenditure [13]. While this equation may on the surface appear simple, there are numerous factors at play, some of which have been well-characterized, while others have yet to be fully elucidated [13].

**Figure 2:**
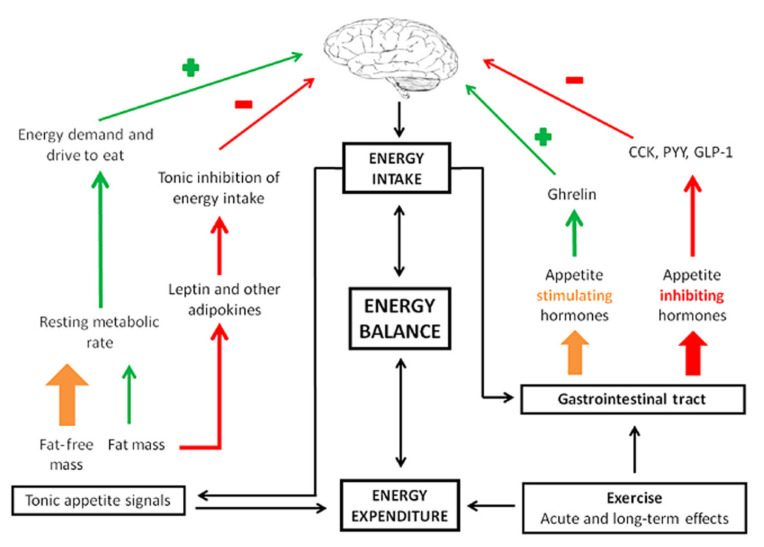
Appetite control. CKK: cholecystokinin; PYY: peptide YY; GLP-1: glucagon-like peptide-1. Reproduced with permission from Blundell et al. [14].

Foundational research from over 50 years ago has identified a non-linear relationship between energy intake, energy expenditure, and energy storage or body weight [13]. Physical activity, including intentional exercise, significantly impacts appetite. In 2015, Blundell et al. published a review discussing the impact of physical activity and exercise on hunger, appetite, and satiety [14]. Appetite and satiety feelings are a result of very complex interactions between the brain and gastrointestinal hormones [15]. Several well-characterized factors influencing appetite are hormones including leptin and ghrelin. Ghrelin, often referred to as the “hunger hormone,” promotes the storage of adipose/fat. Leptin, on the other hand, decreases appetite and helps to regulate body weight. Based upon a review on the underlying neural mechanisms of appetite, Andermann and Lowell described that hormones like leptin and ghrelin communicate with the brain—specifically an area called the hypothalamus [16]. The hypothalamus is responsible for regulating not only appetite but also sleep–wake cycles, mood, and sex drive. If there is an imbalance in leptin or ghrelin, problematic eating behaviors can result, including anorexia or excessive eating [15] (Figure 2).

In 2017, Zanchi et al. published a comprehensive review of the physiology and neuroscientific underpinnings of appetite; they describe “satiety stimulators,” which are hormones such as glucagon-like peptide-1, peptide tyrosine, and cholecystokinin that signal the brain to decrease the drive to eat and cease food intake [17,18,19]. Insulin, a pancreatic hormone, also plays a large role in human metabolism and eating behavior; it is altered for PWCF and in CF-related diabetes [20]. Cortisol is another hormone involved in appetite and there is evidence that higher cortisol levels may be associated with cravings for calorically dense foods [21]. All these hormones interact with the brain; however, other areas outside the hypothalamus are also involved in our appetite and decisions to eat.

The newer field of cognitive neuroscience has advanced the mechanistic understanding of appetite and food motivation. Advances in neuroimaging techniques including functional magnetic resonance imaging have allowed for a better understanding of the brain’s role in appetite and eating decisions. A meta-analysis from 2022 demonstrated that the brain regions responsible for pleasure, reward, taste, homeostasis, deliberate decision- making, and self-regulation are integral for responding to food cues and for making food decisions [22]. In this meta-analysis, the brain regions responsible for regulating appetite were found to be the insula, amygdala, hippocampus, and orbitofrontal cortex [22]. The brain regions responsible for helping to regulate satiety (feeling of fullness) were the hypothalamus, thalamus, caudate, putamen, and anterior cingulate cortex. This review was of healthy adults; there is extremely limited data examining the neurohormonal mechanisms of appetite and satiety among PWCF.

### 1.2. Eating Behaviors and Food Decision-Making

While hormonal levels and brain activations are at play “under the surface,” there are also other factors that influence when, what, and how much we eat. The process of making choices about what to eat and drink is complex and as described above, involves both internal and external influences [23]. Extensive research has demonstrated a host of influences on food choices, including genetic, familial, hormonal, environmental, and physiological determinants [23]. Adults make countless food decisions during just a single day. Across days, weeks, years, and decades, the cumulative impact of patterns of food choices is substantial.

Internal factors include previously mentioned hormones influencing hunger and satiety, the interaction with relevant brain regions, and another important concept called interoception. Interoception refers to a person’s awareness of bodily states including heart rate, body temperature, pain, and hunger [24]. The Embodied Predictive Interoception Coding (EPIC) model purports that, in fact, the anticipation of sensations has an important impact on how we perceive the world. Interoception plays a very important role in a person’s sensations of hunger (appetite) and fullness (satiety), and therefore in eating behaviors and food choices.

Pleasant, delicious foods are referred to as “comfort foods” because of the emotionally appealing aspects of consumption [25]. Mostly in adults, research has shown how important the pleasure centers in the brain, namely the ventral striatum, respond to gustatory stimuli, including sucrose solution and chocolate milkshakes [26]. Eating in the absence of hunger is a related construct used to describe eating past the point of satiety [27]. While there are likely many reasons that people eat in the absence of hunger, a strong body of research has emerged showing that brain-mediated emotional rewards may play a particularly important role in overconsumption [28]. Historically, PWCF may have been instructed to ignore internal cues of satiety and eat past the point of fullness to gain enough calories to maintain or gain weight. Overriding one’s satiety cues can have longstanding and important implications, which may be made more complex as modulators have made the absorption of nutrients and calories easier [29].

Some of the important external influences on feeding and eating behaviors include the following: chest feeding versus formula feeding, parenting, school, peer influence, food marketing, food security, and socioeconomic status [30]. When things go awry in a systematic way and for a sustained period, a person can be considered to have disordered eating behaviors [23].

### 1.3. Historical Nutrition Recommendations for Cystic Fibrosis

The well-known “legacy diet” for PWCF promoted calorically dense, high-fat foods with a high salt content because nutritional status impacted pulmonary function and survival prior to the development of CFTR modulators [31,32,33]. Guidelines encouraged PWCF to maintain a BMI > 50% for children, as well as ≥22 kg/m^2^ and ≥23 kg/m^2^ for adult females and males, respectively. These nutritional benchmarks were shown to be associated with improved pulmonary outcomes [34]. Historically, care teams promoted the legacy diet to the majority of PWCF, especially in the setting of frequent pulmonary exacerbations or declining lung health [3,35]. Many PWCF struggled to even *maintain* their weight prior to CFTR modulators [35].

Given the often substantial changes in their bodies that PWCF may have recently experienced with the ongoing use of CFTR modulators, CF care team nutritional guide- lines are evolving; instead of an emphasis on quantitative BMI cut offs, recommendations are being tailored to promote optimal nutritional health to maintain weight and consume, for example, a fiber-rich diet with lean protein in order to prevent chronic conditions associated with aging such as heart disease [11].

### 1.4. Disordered Eating Behaviors and Eating Disorders

Disordered eating behaviors (DEBs), actions that may pose harm or lead to adverse long-term outcomes, can be an early indication of the development of an eating disorder (ED) [36]. PWCF have unique opportunities for DEBs [11]. Bowel regimens may be misused or overused. In PWCF who are pancreatic insufficient, which is approximately 85% of PWCF, the malabsorption of macronutrients can result from omitting pancreatic enzyme replacement therapy. In fact, pancreatic exocrine insufficiency increases the risk of nutritional abnormalities including fat-soluble vitamin deficiencies, slowed body habitus development, and a low BMI [37,38,39]. CFTR modulators may be restricted, or taken without fat-containing food, as is prescribed for best absorption. PWCF who also have CF-related diabetes and are insulin-dependent may restrict insulin intentionally, which is classified as a diabetes-specific eating disorder (ED-DMT1), colloquially known as diabulimia [40]. Additionally, PWCF may engage in “classic” DEBs such as avoiding/restricting foods or food groups, binge eating, skipping meals, and/or medication misuse [41]. Combining the potential for CF-specific disordered eating, with the more classic behaviors seen in other patient populations, it is evident that there are a multitude of potential actions that can pose harm both acutely and chronically in PWCF [11,41].

Eating disorders (EDs) are serious, complex medical conditions that negatively affect a person’s physical and mental health due to prolonged alterations in eating behaviors. EDs are the second most fatal mental illness, second only to opioid use disorder in the general population [42]. The prevalence of EDs in PWCF is largely unknown. For the general population, at least one in ten people in the U.S. will be diagnosed with an ED in their lifetime, and 22% of children and adolescents worldwide exhibit DEBs [5,43]. In addition, most individuals living with an ED also meet the criteria for another psychological disorder, most commonly anxiety and depression [35,44]. Of women hospitalized for an ED, two-thirds meet the criteria for an anxiety disorder and 90% meet the criteria for depression [6].

ED treatment is essential in reducing morbidity and mortality. Unfortunately, access to treatment is challenging due to treatment cost and a lack of identification of ED symptoms. Identifying individuals with EDs can be difficult, as only 6% of individuals with EDs are in the medically underweight category [45]. In fact, people who are overweight/obese are at higher risk of developing an ED [46,47]. Overall, there is limited identification of EDs, limited insurance coverage of treatment for EDs, and limited access to experts in relation to the treatment of EDs.

### 1.5. Body Image and Weight Stigma

Body image is defined as a multidimensional construct that involves an individual’s internalized view of their body including perceptions, thoughts, feelings, and attitudes to the body’s physical appearance [48]. These thoughts and feelings can comprise physical aspects of weight, figure, thinness, build, strength, sexual attractiveness, physical ability, and aging [48]. The concept of body image is broad; however, research has focused upon how self-perceptions, thoughts, feelings, and attitudes of physical appearance can impact negative psychological consequences [48]. Body dissatisfaction is defined as the negative conceptualization of body image, especially appearance, and is present in all genders and ages. It most frequently develops during adolescence and is more prevalent in females [48,49]. Body dissatisfaction may be further complicated for PWCF due to some visible effects on the body including digital clubbing, scars, altered posture, delayed puberty, and short stature [50]. CF care has traditionally emphasized BMI and weight gain, which may also inadvertently heighten the critical focus of body habitus [31,51].

At one time, undernutrition was a common diagnosis in children with CF and the struggle to maintain adequate weight continued into adulthood [52]. The correlation between improved lung function, survival, and quality of life in PWCF with better nutritional status led to a central focus on body weight, weight gain, and BMI, along with the promotion of the legacy diet that emphasized energy intake over nutrient density [53]. Despite consuming this diet, many PWCF struggled to gain and maintain weight even with additional calories from enteral feeding through bolus or overnight infusions. Notably, PWCF receiving supplemental enteral feeding are generally less satisfied with their body [54]. Gender differences toward body image have been identified for young adults with CF, with females overestimating body size and males underestimating their body size and weight [10,55].

Much of the literature regarding body image and DEBs in PWCF occurred prior to the widespread use of CFTR modulators. The introduction of CFTR modulators may give rise to positive effects on lung and gastrointestinal function, enabling PWCF who had previously struggled to gain and maintain weight to readily gain weight for the first time in their lives [11]. The response to this weight gain in PWCF on CFTR modulators regarding body image has been varied [5]. Some PWCF may feel a combination of perceptions and feelings about their bodies that may be neutral, negative, positive or a mixture of these on any given day, time, or situation. These feelings may range from body dissatisfaction with gradual acceptance and appreciation to body image disturbance, increasing the risk for DEBs and EDs. Notably, the introduction of elexacaftor/tezacaftor/ivacaftor occurred just prior to the COVID-19 pandemic when there was a generalized increase in the occurrence of DEBs and body image disturbances in the general population [56].

A mixed-method study examined the perspectives of adolescents and young adults with CF and their healthcare professionals (HCPs) on body image communication [51]. Although 85% of PWCF reported never discussing body image with a HCP, 74% of HCPs reported discussing this topic. Both PWCF and HCPs agreed that body image is important and should be discussed comfortably and supportively. Kass et al. distributed a one-time web-based survey to a multidiscipline group of CF HCPs in the U.S. via CF Foundation email list management software programs [43]. The survey investigated HCPs’ understanding and perceived importance of DEBs and body image disturbance in adolescent and young adults with CF, along with current screening practices. Most HCPs recognized the importance of screening for DEBs and body image disturbance, but fewer than a third felt comfortable with this process. HCPs reported that assessment tools (86%), standardized partnerships with ED specialists (80%), and CF Foundation or national guidelines (79%) would be helpful to improve screening and counseling [43].

Currently, no evidenced-based treatment for body image disturbances exists. While research is underway, existing approaches include the promotion of body acceptance through body positivity or neutrality and media literacy. To assist PWCF in navigating this evolving landscape, utilizing assessment tools and language that promote positive images of healthy weight gain by using appropriate language like “growing taller, stronger, healthier, well-nourished” (https://uconnruddcenter.org/research/weight-bias-stigma/healthcare-providers/, most recently accessed on 17 August 2025) and examining personal implicit biases toward weight are beneficial. When interacting with PWCF, the focus should be on interventions to improve health rather than weight. Additional resources to support HCPs can be found through the Association for Size Diversity and Health (Table 1), at https://cfreshc.org/SRH-Guide/body-image/, most recently accessed on 17 August 2025), and through the “Tips for CF Care Teams: Conversations about Body Image” from the CF Foundation Mental Health Advisory Committee [57]. Furthermore, caregivers can be encouraged to promote and model apositive body image. Research indicates that early positive body image experiences can have a protective effect against false images from external sources. Caregivers modeling flexible eating, limiting food rules, and demonstrating appreciation of their own body, as well as different sizes and shapes of bodies, can help youth have a healthy body image [58].

### 1.6. Existing DEB Screening Tools

The Sick-Control-One stone-Fat-Food (SCOFF) questionnaire is a simple screening tool used to identify potential EDs (Table 2) [59]. It consists of five questions, each addressing different behaviors or attitudes related to eating and body image. A score of 2 or more affirmative responses suggests a possible ED that warrants further evaluation. It is a quick way to identify people who might need more support for DEBs. The SCOFF questionnaire has been found to be a valid and reliable screening tool for identifying individuals at risk of EDs in the general population. Research indicates that it effectively distinguishes between those who may have an ED and those who do not. Here are some key points about its validity—the SCOFF has a good sensitivity and specificity; it is brief (5 questions); it has been validated in different populations, which supports versatility across cultural contexts; and a positive result does not confirm an ED, rather a comprehensive assessment by a trained professional is necessary for a formal diagnosis.

**Figure 3:**
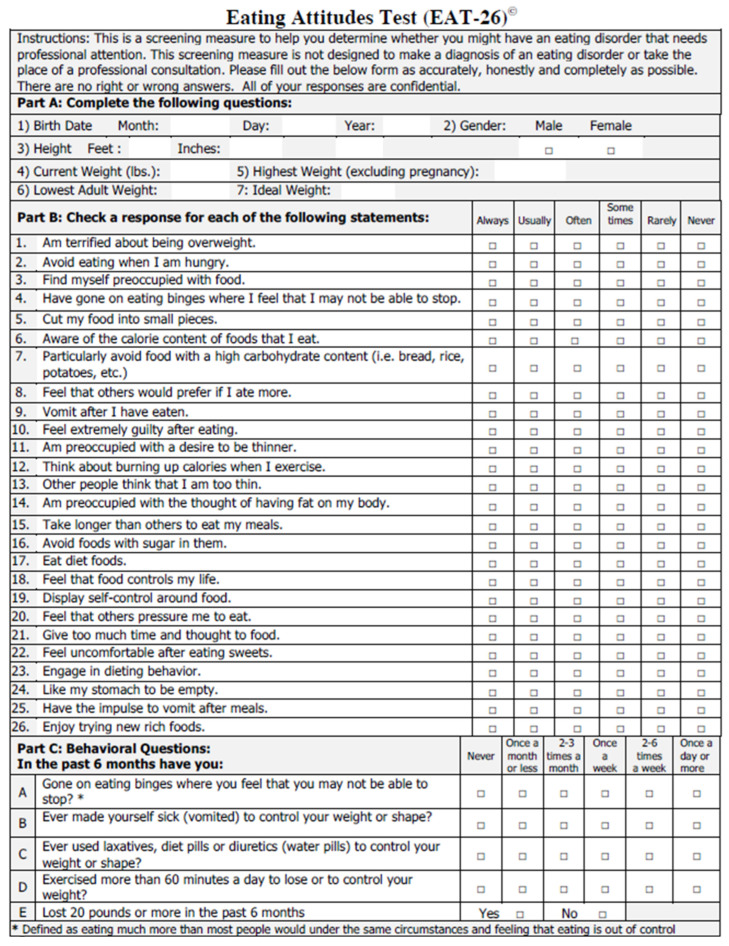
Eating Attitudes Test (EAT-26) questionnaire used in the general population. Reproduced with permission from Garner et al. [60].

The Eating Attitudes Test (EAT-26) is another screening measure designed to identify EDs in the general population that focuses on the following three subscales: dieting, bulimia and food pre-occupation and oral control (Figure 3) [60]. It consists of 26 statements that the respondent indicates a degree of agreement or disagreement with based upon a Likert scale. The EAT-26 also includes five additional behavioral questions regarding maladaptive eating behaviors. The original 26-item version is highly reliable and valid but alone does not yield the diagnosis of an ED. Individuals who score 20 or more on the test should be evaluated by a qualified professional to determine if they meet diagnostic criteria for an ED, as it indicates a high level of concern about dieting, body weight, or maladaptive eating behaviors. The EAT-26 has been especially helpful in assessing ED risk in high school and college general populations, as well as in athletes. While the above two measures have shown promising validity and reliability, they do not include issues that are often relevant for PWCF, making them less-ideal screening tools when applied to PWCF.

The CF-specific screening tool for disturbed eating attitudes or behaviors (CFEAB) questionnaire is a 21-item self-report measure to highlight areas of eating disturbance in PWCF (Table 3). It was administered to 155 PWCF (11–62 years) at Great Ormond Street Hospital in the United Kingdon (U.K.) before the advent of highly effective modulator therapies (HEMTs); it revealed a three-factor structure with good internal consistencies for (1) desire for thinness and weight loss, (2) DEBs, and (3) appetite [61]. Participants respond to the statements in the CFEAB table. Further work to establish construct validity and clarify subscale interpretation was not conducted. Also, this was used prior to the era of HEMTs and may no longer identify maladaptive attitudes towards food, body weight/image, or eating behaviors in PWCF.

### 1.7. Research Gaps and Future Directions

Given the changing landscape of nutrition since the advent of HEMTs, we need a screening tool that is specific to PWCF in the era of HEMTs. We need to survey CF HCPs to hear their voices about what is needed to better assess DEBs and support PWCF experiencing DEBs and EDs. Given that the prevalence and etiology of EDs is largely un- known, interventions designed to reduce known risk factors for EDs and enhance protective factors against the development of DEBs needs to be prioritized. Prior to the widespread use of CFTR modulators, a CF-specific screening tool for assessing DEBs had been developed with promising psychometrics in detecting EDs in the U.K. [61]. To date, there are no reliable and validated screening tools in the U.S. to identify DEBs for PWCF. This contributes to limitations in assessing EDs or DEBs in the CF population. For people with a chronic illness, general ED screening tools have the possibility to miscalculate ED risk [7,10]. Table 4 further characterizes the clinical needs identified for DEBs within the CF population. It is crucial to use a validated screening tool for PWCF to better identify DEBs and body image disturbances to improve accurate identification in PWCF. Currently, questions like, “Do you change your food choices based on your weight?” and “Do you alter your consumption of pancreatic enzymes based on your weight?” can help with screening while a standardized approach is explored. Once identified, a systematic approach to identifying DEBs in PWCF is needed and would likely improve interventions and referral to ED programs when deemed appropriate. CF care team members should be encouraged and supported to seek and complete ED-specific training to enhance their knowledge of the signs, symptoms, and available treatments for EDs.

Parent conversations focused on weight and size are associated with an increased risk of adolescent DEBs [62]; a 2024 systematic review completed by Levinson et al. found a similar correlation in adults [63]. Instead of focusing on weight, the promotion of healthy eating behaviors is protective against DEBs [62]. Healthy eating behaviors include eating regularly throughout the day, consuming a wide variety of foods, and internally regulating eating, which incorporates tuning into hunger and satiety cues.

The focus on healthy eating behaviors promotes a flexible eating style without forbidden foods and avoids dividing foods into “good” versus “bad” categories, which can prevent cognitive distortions linked to EDs [63]. Other behavior interventions such as the promotion of enjoyable physical activity, a reduction in sedentary time, stress management, and body image support may help decrease DEBs. Self-weighing facilitates clinically significant weight loss in those without EDs [63]. It is suggested for individuals with EDs that weekly weighing replaces too-frequent weighing or the avoidance of weighing, as the latter can perpetuate DEBs. While these suggestions exist and may be useful in some cases, it is important to honor and listen to the individual wishes of PWCF. Some PWCF will prefer not to be weighed, nor to discuss their body size at CF clinic encounters. It should also not be assumed that individual PWCF desire to lose weight. HCPs should aim to provide compassionate, patient-centered care, while listening to the individual goals of PWCF [7].

## 2. Limitations and Future Directions

There are some limitations of the current project, one of which is the limited scope of research to draw from. There are a limited number of high-quality studies specifically examining disordered eating in CF populations, with many studies having small sample sizes that may limit generalizability. In addition, the variety and heterogeneity of screening and assessment measures used makes a more-direct comparison across studies quite challenging. As in many areas of research, cross-sectional studies dominate, which can limit the understanding of any causal relationships. There is no standardized screening protocol for disordered eating behaviors in CF care settings and there is an underrepresentation of certain demographic groups (ethnic minorities, adults with CF, those with severe disease progression). Finally, most of the research has been conducted in Western healthcare systems, limiting cross-cultural understanding.

The development and validation of CF-specific disordered eating screening tools and training for healthcare professionals to recognize and address eating concerns in PWCF are vital next steps. Eventually, the design and testing of integrated treatment approaches that address both CF management and eating disorder symptoms will be paramount for improving quality of life for PWCF.

## 3. Conclusions

In this paper, the current information on eating behaviors and disordered eating in PWCF was reviewed and important future directions for the assessment and treatment of disordered eating behaviors to improve quality of life for PWCF were examined. The advent of highly effective CFTR modulators has contributed to weight gain and an evolving nutritional landscape for PWCF [64]. This new and rapidly evolving landscape encompasses an increased risk for issues with body image, relationship with food, DEBs, and, in some cases, EDs. Until more research and standardized interventions can be established, CF HCPs can leverage the multidisciplinary team expertise to utilize more weight-neutral conversations to assess and help address DEBs. In 2018, the CF Foundation Food Security Committee conducted a qualitative survey of the CF community, who identified social workers as a trusted care team member to discuss food insecurity with at that time. This highlights the importance of the multidisciplinary care team approach for discussing complex chronic illnesses that encompass physical and mental health. Registered dietitians, social workers, psychologists, and other care team members are likely already discussing concerns regarding body image, relationship with food, and DEBs with PWCF and their families; therefore, the development of resources and interventions to support care teams and PWCF navigating these challenges with DEBs, body image, and the changing landscape of nutritional recommendations need to be prioritized. To date, there are no reliable and validated screening tools in the United States to identify DEBs for PWCF.

## Figures and Tables

**Table 1:** The Association for Size Diversity and Health.

Weight inclusivity is the ability to accept and respect the inherent diversity of body shapes and sizes and reject the idealizing or pathologizing of specific weights.
Health enhancement is defined as the ability to support health policies that improve and equalize access to information and services and personal practices that improve human well-being, including attention to individual physical, economic, social, spiritual, emotional, and other needs.
Eating for well-being is the capacity to promote flexible, individualized eating based on hunger, satiety, nutritional needs, and pleasure rather than any externally regulated eating plan focused on weight control.
Respectful care acknowledges our biases and works to end weight discrimination, weight stigma, and weight bias. Respectful care provides information and services from an understanding that socioeconomic status, race, gender, sexual orientation, age, and other identities impact weight stigma, as well as support environments that address these inequities.
Life-enhancing movement supports physical activities that allow people of all sizes, abilities, and interests to engage in enjoyable movement, to the degree that they choose.

Reproduced with permission from Ulian et al. [57].

**Table 2:** Sick-Control-One stone-Fat-Food (SCOFF) questionnaire used in the general population.

SCOFF Questionnaire
S: Do you make yourself Sick (vomit) because you feel uncomfortably full?
C: Do you worry you have lost Control over how much you eat?
O: Have you recently lost more than One stone (14 pounds) in a 3-month period?
F: Do you believe yourself to be Fat when others say you are too thin?
F: Would you say that Food dominates your life?

**Table 3:** Cystic Fibrosis Eating Attitudes and Behaviors (CFEAB) questionnaire.

CFEAB Questionnaire
I want to be thinner.
I cut down on food to lose weight.
I am afraid of becoming fat.
I would like to eat less to lose weight.
I spend time wishing I weighed more.
I feel I am too fat.
I exercise as a way to lose weight.
I feel I need to be thin to be happy with myself.
Gaining weight makes me feel happy.
I eat low-fat or low-sugar foods so I won’t gain weight.
The thought of eating food makes me feel worried.
So I won’t gain weight, I deliberately don’t take my enzymes.
So I won’t gain weight, I deliberately don’t take my insulin.
So I won’t gain weight, I deliberately don’t take my extra feeds or supplements.
I make myself vomit (sick) after I eat to control my weight.
I am put off eating because my CF makes me feel sick.
I pretend to others that I have eaten.
I feel guilty after eating.
I enjoy eating.
I feel full quickly.
I have a good appetite for food.

**Table 4:** Clinical needs for disturbed eating in cystic fibrosis.

Clinical Needs	Suggested Approach
Limited understanding of the development of an eating disorder (ED)	Conduct focus groups with CF healthcare providers; conduct focus groups with PWCF
Lack of CF-specific ED screening tools	Development of CF-specific screening tools that address eating disturbances and negative body images; frequent use of screening tools in CF clinics for consistent assessment and early identification
Limited training for healthcare professionals (HCPs) regarding CF-related EDs	Provide education to healthcare providers regarding identifying, addressing, and managing eating disorders; development of resources to provide additional support and education to healthcare providers
Lack of ED prevention programs	Development of a structured prevention program for HCPs to refer patients of concern; collaboration between CF physician, nutritionist, social worker, and psychologist to address patients of concern
Poorly developed ED intervention programs	Development of a structured intervention program that occurs over multiple sessions; treatment approaches that reduce individual, social–familial, and environmental barriers that interfere with self-management

Reproduced with permission from Darukhanavala et al. [10].
